# Relationships between body- and udder-related type traits with somatic cell counts and potential use for an early selection method for water buffaloes *(Bubalus bubalis)*

**DOI:** 10.1093/jas/skad238

**Published:** 2023-07-17

**Authors:** Xinxin Zhang, Kaifeng Niu, Wei Wang, Aftab Shaukat, Xuhong Zhao, Zhiqiu Yao, Aixin Liang, Liguo Yang

**Affiliations:** Key Laboratory of Animal Genetics, Breeding and Reproduction, Ministry of Education, College of Animal Science and Technology, Huazhong Agricultural University, Wuhan 430070, China; International Joint Research Centre for Animal Genetics, Breeding and Reproduction (IJRCAGBR), Huazhong Agricultural University, Wuhan 430070, China; Key Laboratory of Animal Genetics, Breeding and Reproduction, Ministry of Education, College of Animal Science and Technology, Huazhong Agricultural University, Wuhan 430070, China; International Joint Research Centre for Animal Genetics, Breeding and Reproduction (IJRCAGBR), Huazhong Agricultural University, Wuhan 430070, China; Key Laboratory of Animal Genetics, Breeding and Reproduction, Ministry of Education, College of Animal Science and Technology, Huazhong Agricultural University, Wuhan 430070, China; International Joint Research Centre for Animal Genetics, Breeding and Reproduction (IJRCAGBR), Huazhong Agricultural University, Wuhan 430070, China; Key Laboratory of Animal Genetics, Breeding and Reproduction, Ministry of Education, College of Animal Science and Technology, Huazhong Agricultural University, Wuhan 430070, China; International Joint Research Centre for Animal Genetics, Breeding and Reproduction (IJRCAGBR), Huazhong Agricultural University, Wuhan 430070, China; Key Laboratory of Animal Genetics, Breeding and Reproduction, Ministry of Education, College of Animal Science and Technology, Huazhong Agricultural University, Wuhan 430070, China; International Joint Research Centre for Animal Genetics, Breeding and Reproduction (IJRCAGBR), Huazhong Agricultural University, Wuhan 430070, China; Key Laboratory of Animal Genetics, Breeding and Reproduction, Ministry of Education, College of Animal Science and Technology, Huazhong Agricultural University, Wuhan 430070, China; International Joint Research Centre for Animal Genetics, Breeding and Reproduction (IJRCAGBR), Huazhong Agricultural University, Wuhan 430070, China; Key Laboratory of Animal Genetics, Breeding and Reproduction, Ministry of Education, College of Animal Science and Technology, Huazhong Agricultural University, Wuhan 430070, China; International Joint Research Centre for Animal Genetics, Breeding and Reproduction (IJRCAGBR), Huazhong Agricultural University, Wuhan 430070, China; Key Laboratory of Animal Genetics, Breeding and Reproduction, Ministry of Education, College of Animal Science and Technology, Huazhong Agricultural University, Wuhan 430070, China; International Joint Research Centre for Animal Genetics, Breeding and Reproduction (IJRCAGBR), Huazhong Agricultural University, Wuhan 430070, China

**Keywords:** growth curve, healthy udder, milk yield, morphology, nonlinear models

## Abstract

Water buffalo milk is a reliable source of high-quality nutrients; however, the susceptibility of mastitis in buffaloes must be taken into consideration. An animal with somatic cell count (**SCC**) of greater than 250,000 cells/mL is reported to be likely to have mastitis which has serious adverse effects on animal health, reproduction, milk yield, and milk quality. Type traits (**TTs**) of water buffalo can affect SCC in animal milk to some extent, but few reports on the correlation between SCC and TTs are available. In this study, a total of 1908 records collected from 678 water buffaloes were investigated. The general linear model was used to identify factors associated with phenotypic variation of the somatic cell score **(SCS)** trait, including parity, lactation length, calving year, and calving season as fixed effects. Using PROC CORR analysis method, taking calving year and lactation length as covariates, the correlation co-efficient between TT and SCS was obtained. Our results showed that correlation co-efficients between the 45 TTs with SCS ranged from 0.003 to 0.443 (degree of correlation). The correlation between udder traits and SCS was greater than that between body structure traits and SCS. Among udder traits, distance between teats (including front and rear teat distance [*r* = 0.308], front teat distance [*r* = 0.211], and teat crossing distance [*r* = 0.412]) and teat circumference (*r* = 0.443) had the highest correlation with SCS, followed by the leg traits including rear leg height (*r* = −0.354) and hock bend angle (*r* = −0.170). Animal with high rear legs (>48 cm) and short teat crossing distance (<17 cm), and narrow teat circumference (<11 cm) exhibited low SCS. Using four nonlinear models (Von Bertalanffy, Brody, Logistic, and Gompertz), the optimal growth curves of the TTs highly correlated with the SCS (rear leg height and teat crossing distance) were fitted, and the correction co-efficients of these two TTs rear leg height and teat crossing distance of animal from young age (2 mo old) to first lactation (35 mo old) were attained for establishment of early selection method for water buffaloes with low SCS. This study provides theoretical support for early selection of low-SCS water buffaloes and lays a foundation for improving milk quality and promoting healthy development of water buffalo’s dairy industry.

## Teaser

Through the selection of type traits, it is beneficial for us to choose more ideal type (less susceptible to mastitis) water buffaloes.

## Introduction

Water buffalo milk is a rich source of nutrients with a higher content of protein, fat, lactose, total and casein bound calcium than cow's milk (Hussain et al., 2012). According to the latest report from the FAOSTAT (2023) , water buffaloes have produced 137.76 million tonnes of milk in 2021, accounting for 15.00% of the total production that year. Considering this, the research aimed to improve the quality of milk and health in water buffalo will be beneficial to development of the dairy product industry.

Although water buffaloes exhibited multiple advantages such as high milk quality, heat tolerance, and high disease resistance (El Debaky et al., 2019), they are at high risk of developing mastitis, which is an economically damaging problem for water buffaloes worldwide (Guccione et al., 2014). The incidence of subclinical mastitis in water buffaloes is as high as 78% in Nepal (Sah et al., 2020). The economic loss due to mastitis was 15.51 USD per water buffalo per lactation period, with production loss accounting for 49% of the loss and treatment loss accounting for 51% (Singh et al., 2014a). SCC is the most commonly utilized parameter for mastitis detection, with cows being diagnosed with mastitis when their SCC exceeds 250,000 cells/mL in Australia (Ebrahimie et al., 2018).

Some studies have revealed that selection for the type traits **(TTs)** of goats and cows, especially the udder and teat traits contribute to reduction in milk somatic cell count **(SCC)** and mastitis susceptibility (Dube et al., 2009; Rupp et al., 2011). Body structure traits showed moderate genetic correlation with somatic cell score **(SCS**; Dadpasand et al., 2012). Considering low heritability of SCS (0.03 to 0.08; Heringstad et al., 2006, Dal Zotto et al., 2007) and high heritability of udder and teat traits (0.32 to 0.59; Amin et al., 2002), the identification of an udder and teat trait highly correlated with SCS will contribute to selecting water buffalo with low susceptibility to mastitis. Udder traits are an important index for udder health evaluation of dairy cows in Canada (SCS, udder depth, and milking speed), the Netherlands (SCS, udder depth, fore udder attachment, teat length, and milking speed), and Denmark (clinical mastitis, SCS, udder depth, udder support, and dairy form; Miglior et al., 2005). More epidemiological studies are needed to elucidate the effects of TTs on udder health to provide effective strategy for mastitis control in different water buffalo herds and obtain the appropriate economic weights of TTs in mastitis resistance breeding objectives (Hussain et al., 2013).

Fitting the curve of daily milk yield to the day number of lactation based on the growth function provides a more precise description of the productive potential of the cow (López et al., 2015). The establishment of optimal growth curve for Simmental cattle weight for predicting the mature body weight of young animals contributes to the separate management of animal individuals so as to reduce animal feeding costs (Duan et al., 2021). At present, most existing studies focus on fitting growth curve of body weight reflecting meat value and milk yield reflecting milk value in animals. However, no reports on growth curve establishment to fit TTs related to milk quality (represented by SCS) are available, which may lead to uncertainty in early selection of water buffaloes less susceptible to mastitis.

Most water buffaloes are raised on small farm systems in developing countries. Selection of TTs can reduce the SCS value, which compensates for the adverse situation caused by the lack of SCS data in developing countries (Dadpasand et al., 2012). According to our knowledge, most of researches on SCS and TTs have only focused on a few limited udder traits and body structural traits alone, and little attention has been paid to the correlation between multiple TTs and SCS (Dube and Dzama, 2008; Dube et al., 2009; Gibson and Dechow, 2018; Bobbo et al., 2019). In the current research, we explored relationship between 45 TTs and SCS in water buffalo, and the growth curve of TTs highly correlated with SCS. This study will lay a foundation for early selection and breeding of water buffaloes less susceptible to mastitis.

## Materials and Methods

### Experimental animals

Over the period of 2018 to 2022, 678 water buffaloes at Jinniu Animal Husbandry Co. Ltd, Hubei province, China, were studied. A corn silage-based total mixed ration diet was fed to lactating animals in free pens. The average milk yield was 1,130 kg with the lactation length (total days in milking) ranging from 106 to 493 d, and they were milked two times daily. The average SCS was 3.71 with a maximum of 8.98 and a minimum of 0.60. The parity of these animals ranged from one to six. Ethics approval was obtained from Huazhong Agricultural University Animal Care and Use Committee.

### Data

The two datasets used in this study come from a total of 678 water buffalo. Dataset 1 is from lactating water buffaloes (*N* = 155) for correlation analysis of SCS and TTs. The 45 TTs including 33 body structure traits and 12 udder traits, of which body structure consisting of body weight, 18 body capacity traits (heart girth, leg rump circumference, chest width, loin strength, waist length, neck length, back length, chest depth, body depth, waist angle width, chest meat ball width, wither height, back height, waist angle height, rump height, hip height, body length, body oblique length), four rump traits (anovaginal distance, rump angle, rump width, and rump length), seven foot and leg traits (heel depth, cannon bone circumference, left front hoof circumference, front leg height, rear leg height, foot angle and hock bend angle), three head traits (head length, nose width, forehead width); and udder traits consisting of front udder angle, central ligament, udder depth, teat length, front teat distance, front and rear teat distance, teat crossing distance, rear teat distance, teat circumference, udder circumference, rear udder height, and rear udder width. Dataset 2 was used to establish growth curves of TTs highly correlated with SCS, consisting 1,908 records of 678 water buffaloes aged 0 to 124 mo in the growth curve study, 1,206 records of 33 body structures and 702 records of 5 udder traits (including teat crossing distance, front and rear teat distance, front teat distance, rear teat distance, and teat length). The remaining seven udder traits could not be measured because the udder of calf was not developed yet. The measurement time of each trait was concentrated in midlactation, and the specific measurement method was the same as in our previous report (Zhang et al., 2022).

The milk samples were tested for SCC with a milk component detector (CombiFoss FT + FOSS Analytical Hillerød, Denmark) in Hubei Center of Dairy Herd Improvement (**HCDHI**) on the monthly basis. The SCC was transformed into SCS according to the following formula: SCS = 3 + log_2_(SCC/100,000) for normality and homogeneity of the SCC. Lactation SCS referred to the average of all the monthly test-day SCS values during entire lactation (Rupp and Boichard, 2000; Berry et al., 2007; Bobbo et al., 2018).

### Statistical analysis

#### Factors affecting somatic cell score

A general linear model (version 9.4, SAS Institute Inc., Cary, NC) was used to determine the effect of lactation length, ­parity, calving year, and calving season on SCS (Nyamushamba et al., 2014). The model formula was as follows:


$$\begin{array}{l} y_{\text{ijkl}}\;=\;\mu \;+\;\text{Lactation ​​}\;\text{​​}{\text{ length}}_{i}\;+\;{\text{Parity}}_{j}\;+\;\text{Calving ​​}\;\text{​​}{\text{ season}}_{k}\;\\ +\;\text{Calving ​​}\;\text{​​}{\text{ year}}_{l}\;+\;{\left(\text{Lactation ​​}\;\text{​​ length}\;\times \;\text{Parity}\right)}_{\text{ij}}\;\;+\;e_{\text{ijkl}}, \end{array}$$

where *y*_ijkl_ is the SCS. μ is overall population mean. Lactation length_*i*_ is the *i*th fixed effect (*i* = 1 to 3; category 1, lactation length ≤ 200; category 2,201 ≤ lactation length ≤ 270; and category 3, lactation length ≥ 271). Parity_*j*_ is the *j*th fixed effect (*j* = 1 to 3; category 1 = 1; category 2 = 2, and category 3 = 3 to 6). Calving season_*k*_ is *k*th fixed effect (k = 1 to 4; category 1 = Spring, including March to May; category 2 = Summer, including June to August; category 3 = Autumn, including September to November; category 4 = Winter, including January to February, and December). Calving year_*l*_ is *l*th effect (l = 1 to 5, category 1 = year 2017, category 2 = year 2018, category 3 = year 2019, category 4 = year 2020, category 5 = year 2021). (Lactation length × Parity)_*ij*_ is the interaction effect between lactation length and parity. *e*_ijkl_ is the random residual.

### Correlation analysis between type traits and somatic cell score

The PROC CORR procedure in SAS was utilized to conduct correlation analysis. Considering the significant impact of lactation length and calving year on SCS, we incorporated them as covariates into the correlation analysis between TT and SCS. The Wilcoxon-test in R software was employed to compare SCS values across different TT ranges.

### Optimal growth curve of the type trait highly correlated with somatic cell scores

The four most widely used nonlinear models (Von Bertalanffy, Brody, Logistic, and Gompertz; Gbangboche et al., 2008) were used to establish growth curves for TTs affecting SCS from young to adult of age. The formulae of four nonlinear can be specified as:

Von Bertalanffy,


$$y=A*{\left(1-B*{\text{exp}}^{-K*t}\right)}^{3},$$

Brody,


$$y=A*\left(1-B*{\text{exp}}^{-K*t}\right),$$

Logistic,


$$y=\frac{A}{\left[1+B*{\text{exp}}^{-K*t}\right]},$$

Gompertz,


$$y=A*{\text{exp}}^{\left(-B*{\text{exp}}^{-K*t}\right)},$$

in which *Y* represents the TTs value of water buffaloes at age *t*; *A* represents mature TTs; *B* represents an integration constant related to initial animal TTs; *K* is the maturity rate, which is interpreted as a relationship between TTs change and mature TTs to indicate how quickly the animal is approaching its adult TTs; *t* is the age in month; *exp* is the natural base logarithm.

The PROC NLMIXED program using SAS software was used to fit the growth curve of TTs highly correlated with SCS, and the parameters of the models were estimated by the dual quasi-Newton as the optimization technique (Goldberg and Ravagnolo, 2015). The model fitting degree with the smallest values of −2 log-likelihood, Akaike information criterion (**AIC**), Bayesian information criterion (BIC), and the highest *R*^2^ was considered as the best model, and the corresponding growth curve of the TTs was considered as the optimal one.

## Results and Discussion

The statistical descriptions of TTs are based on our earlier findings (Zhang et al., 2022). The average (SD) of body weight, wither height, and body depth was 615.67 (80.28) kg, 139.43 (4.50) cm, and 81.29 (5.86) cm, ranged from 405.00 to 829.50 kg, 129.00 to 150.00 cm and 67.00 to 94.00 cm, respectively. Average (SD) rear leg height was 47.37 (1.91) cm, ranged from 42.80 to 52.00 cm. Teat circumference and teat crossing distance ranged from 7.00 to 14.30 cm and 8.75 to 24.00 cm with a mean (SD) of 10.43 (1.58) cm and 15.72 (3.09) cm, respectively.

### Factors affecting the somatic cell score

Our study showed that parity (*P* = 0.1319), calving season (*P* = 0.1190), and parity × lactation length (*P* = 0.8221) had no significant effect on SCS, while lactation length (*P* = 0.0465) and calving year (*P* = 0.0055) significantly affected SCS. The SCS in milk may be influenced by multiple factors, however, the impact of these factors on SCS could vary based on previous studies. Calving season did not have a significant impact on SCS, whereas calving year was identified as a crucial factor influencing the variation of SCS (Rekik et al., 2008), which is consistent with our research findings. However, Wu et al. (2021) reported that SCS tends to be higher during summer. In contrast to our findings, Yang et al. (2013) reported that SCS increased with parity, while Alhussien and Dang (2018) observed that there was no significant difference in the milk SCC between cows with primiparous and cows with 2-4 parity.

### Correlation between type traits and somatic cell score

Our result showed correlation co-efficients between the 45 TTs and SCS ranging from 0.003 (between udder circumference and SCS) to 0.443 (between teat circumference and SCS). SCS was significantly positively correlated with the four TTs: teat circumference (*r* = 0.443, *P* < 0.0001), teat crossing distance (*r* = 0.412, *P* < 0.0001), front and rear teat distance (*r* = 0.308, *P* = 0.0001), and front teat distance (*r* = 0.211, *P* = 0.0088). SCS was significantly negatively correlated with the five TTs: rear leg height (*r* = −0.354, *P* < 0.0001), hock bend angle (*r* = −0.170, *P* < 0.05), loin strength (*r* = −0.218, *P* = 0.0068), central ligament (*r* = −0.160, *P* < 0.05), and front udder angle (*r* = −0.186, *P* < 0.05). TTs can often be used as predictors of the mastitis incidence, but the relationship between TTs and SCS varies with cattle breeds, and thus different TTs are required to be identified for each breed (Seykora and McDaniel, 1985; Dadpasand et al., 2012).

The correlation between SCS and most TTs was low, which was in agreement with the findings by Gibson and Dechow (2018). Our results were in line with one previous report that better udder (including teat) and leg conformation were associated with low SCS (Stefani et al., 2018). Singh et al. (2014b) observed that cows with teats situated closer to the floor were more susceptible to mastitis, which could explain the negative correlation between rear leg height and SCS found in the current investigation. A lower height of the rear legs is associated with an increased likelihood of udder contamination by environmental pathogens due to soiling. Similar to our results, Chrystal et al. (1999) and Zwertvaegher et al. (2013) reported that the mastitis incidence cases could be reduced by eliminating animals with large teat diameter during selection. The size of the teat canal and sphincter patency play crucial roles in preventing bacterial invasion into the teat cistern. However, both a wider teat canal and a sphincter that does not effectively close the teat orifice are associated with a wider teat diameter (Seykora and McDaniel, 1985). Earlier research by DeGroot et al. (2002) and Seykora and McDaniel (1986) supported the current findings by demonstrating that cows with shorter distances between teats had lower SCC. The effect of udder traits on SCS was greater than that of body structure traits, which was consistent with previous reports (Dadpasand et al., 2012).

**Figure 1.**
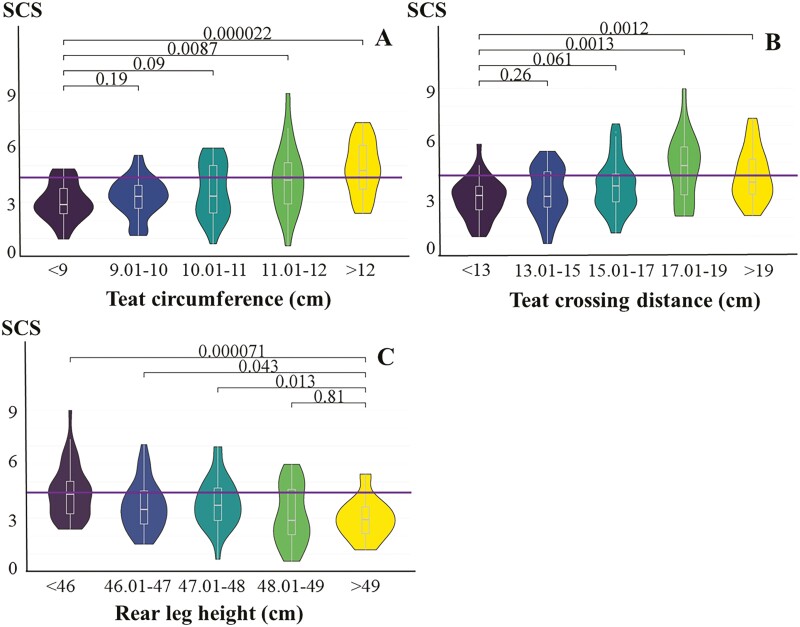
SCS values at different levels of TTs in water buffaloes by Wilcoxon test. Note. The purple line indicates the number of SCC at 250,000 cells/mL; (A) SCS at five levels of teat circumference. (B) SCS at five levels of teat crossing distance. (C) SCS at five levels of rear leg height.

The values of SCS at different levels of teat circumference, teat crossing distance, and rear leg height are shown in Figure 1. SCS increased with the increase of teat circumference, and SCC was close to 250,000 cells/mL (SCS = 4.32) when teat circumference was greater than 11 cm. For a teat crossing distance of less than 15 cm, low SCS values were observed, whereas the SCS was significantly elevated at a teat crossing distance of greater than 17 cm. SCS value gradually decreased with the increasing rear leg height, and SCS value was the lowest when rear leg height was greater than 48 cm. Taken together, high rear legs (>48 cm), short teat crossing distance (<17 cm), and narrow teats circumference (<11 cm) were associated with low SCS in the water buffaloes.

### Establishments of optimal growth curves for type traits highly correlated with somatic cell score

In this study, teat circumference, teat crossing distance, and rear leg height exhibited the greatest value in reducing the incidence of mastitis, but the low degree of development at the calf stage made teat circumference unmeasurable, and thus we fitted optimal growth models only for teat crossing distance and rear leg height. Table 1 shows the parameters *A*, *K*, *B*, AIC, BIC, −2 log-likelihood, and *R*^2^ value corresponding to two traits (rear leg height and teat crossing distance) in the four nonlinear growth models. The *R*^2^ values ranged from 0.6124 to 0.6467, which were comparable to the nonlinear model describing cattle scrotal circumference growth (0.67 to 0.69) as reported by Loaiza-Echeverri et al. (2013), but inferior to those of body weight growth (0.951 to 0.954) as reported by Duan et al. (2021). Body weight growth exhibited greater regularity and was more amenable to modeling.

**Table 1.** Parameter and goodness of fit for four nonlinear models of water buffalo rear leg height and teat crossing distance

Trait	Model	Parameters			Values of criteria			
		*A* ^a^	*K* ^b^	*B* ^c^	−2 log- likelihood	AIC^d^	BIC^e^	*R* ^2^
Rear leg height	Logistic	46.5460	0.1954	0.3252	5,618.3	5,626.3	5,646.6	0.6467
	Von Bertalanffy	46.5773	0.1755	0.0906	5,618.1	5,626.1	5,646.5	0.6467
	Brody	46.5947	0.1658	0.2490	5,618.3	5,626.3	5,646.7	0.6466
	Gompertz	46.5691	0.1804	0.2839	5,618.1	5,626.1	5,646.4	0.6467
Teat crossing distance	Logistic	15.1360	0.0502	1.7577	3,392.3	3,400.3	3,418.5	0.6124
	Von Bertalanffy	15.6326	0.0332	0.3067	3,390.0	3,398.0	3,416.3	0.6136
	Brody	16.0783	0.0253	0.6871	3,390.5	3,398.5	3,416.7	0.6134
	Gompertz	15.4736	0.0373	1.0735	3,390.3	3,398.3	3,416.5	0.6135

^a^Mature value,^b^Maturity rate,^c^Time-scale parameter,^d^Akaike information criterion,^e^Bayesian information criterion.

**Figure 2.**
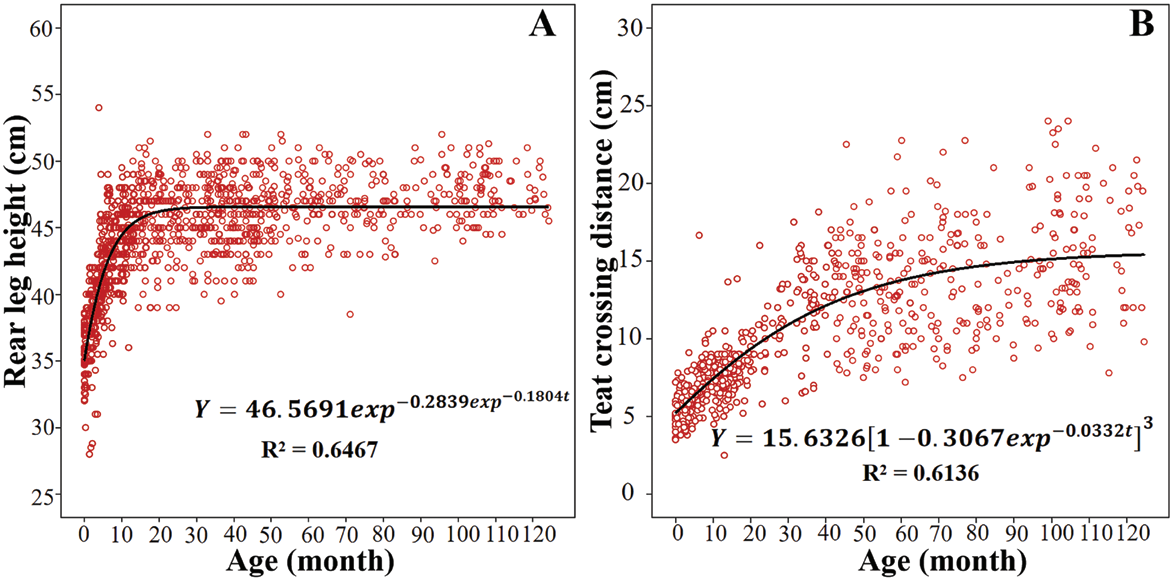
Optimal growth curve for rear leg height fitted by Gompertz model and for teat crossing distance fitted by Von Bertalanffy model in water buffaloes. Note. (A) Rear leg height. (B) Teat crossing distance. Observed values are shown in red; and predicted values are shown in black.

We compared the four models and found that their differences in the −2 log-likelihood, AIC, BIC, and *R*^2^ was very small. All the four models described the growth curve of rear leg height and teat crossing distance well. Following the principle that the better the model, the smaller the −2 log-likelihood, AIC, and BIC, the higher the *R*^2^, the Gompertz model was adopted to describe growth curves for rear leg height, and the Von Bertalanffy model was employed to elucidate growth curves for teat crossing distance (Table 1; Figure 2). Understanding the biological implication of model parameters and their relationships helps to develop breeding strategies and modify the shape of growth curves (Podisi et al., 2013). The mean maturity values for rear leg height and teat crossing distance were 46.5718 ± 0.0202 and 15.5801 ± 0.3914, and the mean maturity rate (growth rate of each trait) were 0.1793 ± 0.0123 and 0.0365 ± 0.0104 in the four models, respectively.

**Figure 3.**
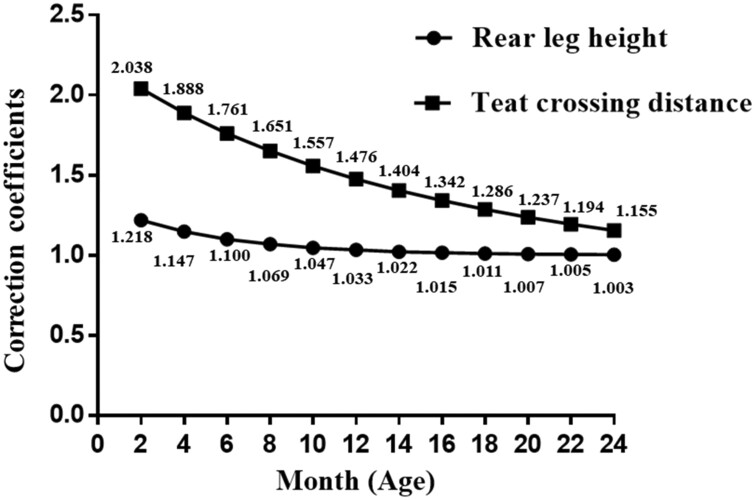
Correction co-efficients for rear leg height and teat crossing distance traits in water buffaloes from young age to first lactation (35 mo old) with rear leg height fitted by model Gompertz and teat crossing distance fitted by model Von Bertalanffy.

Establishing growth curves for TTs related to the commercial life of animals allows economical prediction of production and breed growth characteristics improvement by using curve parameters as selection criteria (Lupi et al., 2015). The growth curve for body weight of northern Brazil beef cattle was used to predict the weight of the mature animal, and thus animals with poor growth potentials were eliminated before their maturity (Lopes et al., 2012). For the milk production animals, it is more meaningful to fit the growth curve related to milking performance. Water buffalo commences first lactation at approximately 35 mo of age (Eldawy et al., 2021). This study obtained correction co-efficients for the TTs highly correlated with SCS by fitting optimal growth curves for these TTs of water buffaloes from young age (2 mo old) to first lactation (35 mo old) with rear leg height fitted by model Gompertz and teat crossing distance by model Von Bertalanffy (Figure 3), which can contribute to selecting water buffaloes with healthy udders and high milk quality in advance.

## Conclusion

By estimating the correlation between TTs and SCS, this study identified the importance of several TTs in udder health and improving milk quality in buffalo. Water buffaloes with high rear legs, short teat crossing distance, and narrow teat circumference were less prone to mastitis. Therefore, we should take these traits into full consideration when breeding buffalo with better performance. The nonlinear model used to fit the SCS-related TT growth curve and establish an early prediction method of TT value could help cull mastitis susceptible calves in advance and help to reduce the incidence of mastitis in water buffalo.

## Abbreviations:

*A*, mature value;AIC, Akaike information criterion;*B*, time-scale parameter;BIC, Bayesian information criterion;*K*, maturity rate;SCC, somatic cell count;SCS, somatic cell score;TT, type trait
